# Oral Administration with Recombinant Attenuated Regulated Delayed Lysis *Salmonella* Vaccines Protecting against *Staphylococcus aureus* Kidney Abscess Formation

**DOI:** 10.3390/vaccines10071073

**Published:** 2022-07-04

**Authors:** Yanchen Liang, Haochi Zhang, Na Pan, Yang Liu, Shouxin Sheng, Haotian Li, Xuemei Bao, Xiao Wang

**Affiliations:** State Key Laboratory of Reproductive Regulation and Breeding of Grassland Livestock, School of Life Science, Inner Mongolia University, Hohhot 010070, China; liangyanchen21@mail.imu.edu.cn (Y.L.); 21908028@mail.imu.edu.cn (H.Z.); 22008032@mail.imu.edu.cn (N.P.); 21808021@mail.imu.edu.cn (Y.L.); shouxins1230@mail.imu.edu.cn (S.S.); 31908093@mail.imu.edu.cn (H.L.); 31908096@mail.imu.edu.cn (X.B.)

**Keywords:** *Staphylococcus aureus*, attenuated *Salmonella*, oral vaccines, rEsxAB, rHla_m_

## Abstract

Abscess formation is one of the main symptoms of *Staphylococcus aureus* infection. It is very important to inhibit abscess formation for preventing *S. aureus* persistent infection. To find a feasible solution, the live oral vaccines delivering *S. aureus* antigens, rEsxAB and rHla_m_, were constructed, which were based on the attenuated regulated delayed lysis *Salmonella enterica* subspecies Serovar Typhimurium strain χ11802, and the inhibiting effect on abscess formation was evaluated in mice kidneys. As the results showed, after oral administration, humoral immunity was induced via the mucosal route as the antigen-specific IgG in the serum and IgA in the intestinal mucus both showed significant increases. Meanwhile, the production of IFN-γ and IL-17 in the kidney tissue suggested that Th1/Th17-biased cellular immunity played a role in varying degrees. After challenged intravenously (i.v.) with *S. aureus* USA300, the χ11802(pYA3681−*esxAB*)-vaccinated group showed obvious inhibition in kidney abscess formation among the vaccinated group, as the kidney abscess incidence rate and the staphylococcal load significantly reduced, and the kidney pathological injury was improved significantly. In conclusion, this study provided experimental data and showed great potential for live oral vaccine development with the attenuated regulated delayed lysis *Salmonella* Typhimurium strains against *S. aureus* infection.

## 1. Introduction

*Staphylococcus aureus*, a commensal pathogen, is acquired both in the community and in hospitals [1]. *S. aureus* causes a wild range of diseases, from mild to life-threatening infections [1,2]. Abscess formation makes *S. aureus* infections persistent [3]. Additionally, it seems that kidney abscess is an important symptom of *S. aureus* infection [4,5,6]. With the abuse of antibiotics, the multidrug-resistant *S. aureus* strains continue to emerge and make it much more difficult to clinically treat infection, especially methicillin-resistant *S. aureus* (MRSA) and vancomycin-resistant *S. aureus* (VRSA) [7,8]. Therefore, the protective vaccine is a promising choice.

In recent years, virulent factors have shown potential as subunit vaccines against *S. aureus* infection, which present effective protection [9,10]. Among them, EsxA, EsxB, and Hla are promising candidate vaccines for inhibiting abscess formation [6,11]. EsxA and EsxB are secreted via the *S. aureus* ESAT-6 secretion system and play important roles in *S. aureus* murine abscess formation [12]. Their sequences remain highly conserved in the genome among different *S. aureus* strains [13]. What is more, when used as subunit vaccines and used to vaccinate mice by intraperitoneal (i.p.) injection, EsxA and EsxB both enable to induce Th1- and Th17-biased cellular immunity, which plays a major part in inhibiting *S. aureus* infection [14]. As Charalampia et al. reported, mice were challenged with *esxA* and *esxB* mutant *S. aureus* strains and showed a defect in abscess formation [12]. Hla (α-hemolysin) is a pore-forming cytotoxin binding to most eukaryotic cells and is possibly relevant to the pathogenesis of sepsis, pneumonia, and severe skin infection [15]. Hla_H35L_ is an attenuated Hla mutant and was used to protect against *S. aureus* infections in recent studies [11,16]. As Adam et al. reported, Hla_H35L_ showed its ability to moderate the severity of *S. aureus* skin infections and inhibited the abscess formation [11]. Furthermore, administration with 4C-Staph/alum including EsxAB (EsxA and EsxB fused for expression) and Hla_H35L_ exhibits protective efficacy in four murine models, including kidney abscess, peritonitis, skin, and pneumonia models [6]. In our previous study, another Hla mutation (Hla_m_) was chosen, with the histidine mutated to leucine at the 285th aa, as a subunit vaccine. It also showed high protection in *S. aureus* lethal infection (data not shown). In this research, EsxAB and Hla_m_ were chosen as delivered *S. aureus* antigens.

Live attenuated *Salmonella enterica* subspecies serovar Typhimurium strains (hereafter *Salmonella* Typhimurium) have been developed as delivery vectors, carrying heterologous antigens and used for immunization via the mucosal route [17,18,19]. There are several good traits with the recombinant attenuated *Salmonella* Typhimurium strains. Firstly, oral administration with live recombinant attenuated *Salmonella* Typhimurium strains provides needle-free, low-cost protection against disease, and the strains will survive a period under the stress of GI trace [19,20]. At the same time, the strains will elicit long-lasting protection with mucosal and cell-mediated immune responses, against a range of pathogens [21,22]. Secondly, they can also invade and colonize the deep effector lymphoid tissues, which is essential for inducing memory T cells [23]. Thirdly, the balanced-lethal system design of the live attenuated *Salmonella* Typhimurium strains eliminates the drug-resistance markers, and the plasmid vector can be stably maintained with heterologous antigen expression [24]. Fourthly, *Salmonella* Typhimurium outer membrane proteins, flagellin, and its ghosts have been developed as adjuvants enhancing antigen-specific immune response [25,26,27]. Moreover, the regulated delayed lysis system offered another means for attenuation and added safety features to confer biological containment. This system allows attenuated *Salmonella* Typhimurium strains to colonize in the tissue only if the *araC* P_BAD_ promoter is activated with an exogenous source of arabinose supplied [28]. After the cells of attenuated *Salmonella* Typhimurium strains replicate for 6–10 rounds, the arabinose accumulated within the bacterial cells will be exhausted [29]. At the same time, the C2-regulated promoter, P22 P_R_ will work and antisense mRNA synthesis will block the residual mRNA of *asdA* and *murA*, which encode the essential enzymes for the bacterial cell wall [28]. These concerted activities eventually result in cell lysis. The details of this system have been described elsewhere [28,30]. 

In this study, attenuated regulated delayed lysis *Salmonella* Typhimurium strain χ11802 was utilized as an antigen delivery vector, expressing *S. aureus* EsxAB (rEsxAB) and Hla_m_ (rHla_m_), respectively. This strategy emphasized the T-cell-mediated immunity of the oral vaccines. After BALB/c mice were orally administrated with these recombinant attenuated *Salmonella* Typhimurium strains, and then challenged with *S. aureus* USA300 by i.v., the inhibition efficacy of kidney abscess formation was evaluated. As a result, among all the vaccinated groups, the χ11802(pYA3681−*esxAB*)-vaccinated group showed obvious inhibition in kidney abscess formation. These results showed the great potential for the live oral vaccine development with the attenuated regulated delayed lysis *Salmonella* Typhimurium strains against *S. aureus* infection.

## 2. Materials and Methods

### 2.1. Mice and Ethics Statement

The 6–8-week-old specific-pathogen-free (SPF) female BALB/c mice were purchased from Beijing Vital River Laboratory Animal Technology Co., Ltd. (Beijing, China), and fed under SPF conditions. All animal-related experimental protocols applied in this study were conducted under the standards of the Ethics Committee of Inner Mongolia Medical University (SCXK2016-0001).

### 2.2. Bacterial Strains and Growth Conditions

Attenuated regulated delayed lysis *Salmonella* Typhimurium strain χ11802 and *Escherichia coli* strain χ6212 were kindly provided by the research team of Dr. Roy Curtiss III, together with the pYA3681 plasmid. All the strains and plasmids are listed in Table 1. χ11802 and χ6212 were cultured in Luria–Bertani (LB) broth or on an agar plate under aerobic condition at 37 °C, both needed supplementing with 50 μg/mL Diaminopimelic acid (DAP), and 0.02% *w*/*v* arabinose was additionally needed for χ11802 growth. *S. aureus* USA300 was reserved in our laboratory and cultured in LB broth or agar plate at 37 °C under aerobic conditions overnight.

### 2.3. Construction of rHla_m_ and rEsxAB Delivery Vector Based on Attenuated Regulated Delayed Lysis Salmonella Typhimurium Strain χ11802

The ESAT-6 secretory proteins, EsxA and EsxB, were merged with a flexible linker, (GGGGS)_3_. Then, *S. aureus* antigen genes, the *esxAB,* and the *hla*_m_, were optimized and synthesized into cloning vector pUC57, respectively, by GenScript Biotech (Nanjing, China), according to the codon bias of *Salmonella* Typhimurium. Then, the *bla*_SS_ (β-lactamase type II signal sequence) together with the HA-tag fragment was fused to *esxAB* and *hla*_m_ for heterologous protein secretion [31]. Antigen gene fragments in pUC57 and linearized pYA3681 plasmid vector were harvested by being digested with restriction enzymes, *Nco*I and *Sma*I. The antigen gene fragments were fused into pYA3681 with T4 DNA ligase. Then, the recombinant plasmids were introduced into competent χ6212 by the heat-shock method, and the bacteria cells were coated on the LB agar plate and cultured overnight at 37 °C under aerobic conditions. The colonies were inoculated into the LB broth for recombinant plasmid harvest. After the plasmids were double-enzyme digested (*Nco*I and *Sma*I) and sequenced for verification, they were finally introduced into χ11802 competent cells, and the recombinant strains of χ11802 were grown on the LB agar plate containing 0.2% *w*/*v* arabinose under the same condition as described above. Positive clones were recognized by double-enzyme digestion and sequencing of the recombinant plasmids in χ11802. All recombinant strains were then stored at −80 °C for later experiments.

### 2.4. SDS-PAGE and Western Blot

SDS-PAGE and Western blot were conducted following standard methods. After recombinant strains of χ11802 were induced by Isopropyl-β-D-thiogalactopyranoside (IPTG, 1 mg/mL), pellets were harvested by centrifugation at 5000× *g*, then resuspended with phosphate-buffered saline (PBS) solution. The secreted proteins in the medium were 20-fold concentrated by Trichloroacetic Acid (TCA) precipitation and washed 3 times with PBS solution. Bacterial pellets were ultrasonic crashed at a low temperature. All samples were mixed with pre-stained protein-loading buffer, boiled for 10 min, and analyzed by 12% SDS-PAGE. Protein samples in the polyacrylamide gel were transferred to the 0.22 μm PVDF membrane and blocked for 1 h at room temperature with 5% *w*/*v* skimmed milk/TBST solution (tris buffered saline solution containing 0.2% *v*/*v* Tween-20). PVDF membranes were incubated at 4 °C overnight with mouse anti-HA tag monoclonal antibody (Proteintech; Rosemont, IL, USA, used at 1:10,000). After washing 3 times with TBST solution, polyvinylidene difluoride (PVDF) membranes were incubated with HRP-conjugated goat anti-mouse IgG secondary antibody (Proteintech; Rosemont, IL, USA, used at 1:5000) for 1 h at room temperature. Then, the membranes were washed 3 times with TBST as before. The protein bands were visualized with ECL Western blotting substrate (Thermo Fisher Scientifics, Waltham, MA, USA) and the signal was analyzed by the ChemiScope chemiluminescence imaging system (CLINX, Shanghai, China).

### 2.5. The Dependence of Recombinant Attenuated Regulated Delayed Lysis Salmonella Typhimurium Strains on Arabinose In Vitro and In Vivo

Recombinant attenuated regulated delayed lysis *Salmonella* Typhimurium strains, χ11802(pYA3681−*esxAB*) and χ11802(pYA3681-*hla*_m_), were streaked on the LB agar plate with or without 0.02% *w/v* arabinose, incubated at 37 °C overnight. Then, the single bacterial colony of recombinant strains on the LB agar plate (with arabinose) was inoculated into LB broth (without arabinose), as the first generation. Then, the first generation was subcultured with a series of gradient concentrations, 1:10, 1:100, and 1:1000 *v*/*v*, as the second generation. If there was no obvious growth inhibition, bacteria from 1:10 subcultured group was subcultured again with a series of gradient concentrations, 1:10, 1:100, and 1:1000 *v*/*v*, as the third generation. This step would be repeated until obvious bacterial growth inhibition appeared. At the same time, the recombinant strains of χ11802 from the LB broth (without arabinose) or 1:10 subcultured group were also subcultured in LB broth with 0.02% *w*/*v* arabinose as the positive control.

After deprivation of food and water for 5 h, the BALB/c mice were each orally administrated with ~3 × 10^8^ CFU recombinant strains of χ11802, which expressed EsxAB or Hla_m_ and were resuspended in 100 μL PBS solution. These strains were cultured in LB broth with 0.02% *w*/*v* arabinose, centrifuged, and resuspended with PBS solution. Then, 2 h later, food and water were returned to the mice. On days 1, 7, 14, and 28 after vaccination, the intestine, stomach, mesenteric lymph nodes (MLN), spleen, and Peyer’s Patches were collected from every 3 mice for the bacterial loading evaluation. All tissues were homogenized, and the numerical difference in bacterial colonies on a selenite cystine agar plate with or without 0.02% *w*/*v* arabinose was regarded as the number of arabinose-dependent strains.

### 2.6. BALB/c Mice Orally Administrated with Recombinant Attenuated Regulated Delayed Lysis Salmonella Typhimurium Strains

BALB/c mice were randomly divided into 4 groups, initially. There were 19 mice in each group and orally administrated at the same time with the PBS solution, χ11802(pYA3681), χ11802(pYA3681−*esxAB*), or χ11802(pYA3681−*hla*_m_). Each of the strains were ~3 × 10^8^ CFU resuspended in 100 μL PBS solution. Two weeks after primary immunization, all groups were booster immunized. Nine in each group were used for evaluating the antigen-specific IgA in the intestinal mucosa, the Interferon-γ (IFN-γ) and interleukin 17 (IL-17) in the tissue of the kidneys and spleens. The other mice, whose serum-specific IgG was measured by enzyme-linked immunosorbent assay (ELISA), were utilized for *S. aureus* USA300 challenge experiments. The food and water were withdrawn and returned as previously described.

### 2.7. rEsxAB- and rHla_m_-Specific IgG Level in the Serum Measured by ELISA

Sera were collected on day 14 after primary and booster immunization, respectively. Blood samples were incubated at 37 °C for 2 h and centrifuged for serum. The sera were stored at −20 °C for later detection. The 96-well MICROLON ELISA plates were coated with 100 μL protein diluent (5 μg/mL rEsxAB or rHla_m_ protein, extracted in the previous study), and placed at 4 °C overnight. Then, the plates were washed 3 times with PBST (PBS with 0.05% *v*/*v* Tween-20) solution, blocked with 5% BSA at 37 °C for 2 h, and further washed 3 times with PBST solution. Corresponding to the coated protein, 100 μL/well diluted sera (1:100 dilution with PBS solution) were added, and the plates were incubated at 37 °C for 2 h. After washing with PBST solution 5 times, HRP-conjugated goat anti-mouse IgG (Proteintech) solution (1:2000 dilution with 1% BSA) was added to the wells, and the plates were incubated at 37 °C for 2 h. Finally, after washing another 5 times with PBST solution, 100 μL/well TMB single-component substrate (Solarbio, Beijing, China) solution was added, with the incubation at 37 °C for 2 h. Then, 50 μL/well ELISA stop solution (Solarbio, Beijing, China) was added, and absorbance was measured at OD_450_.

### 2.8. rEsxAB- and rHla_m_-Specific IgA in the Mouse Intestinal Mucosa Measured by ELISA

Intestinal mucus was collected on day 7 after booster immunization. Each sample (each ~0.05 g) was suspended with 500 μL PBS buffer. After centrifuging, the supernatant was collected and then stored at −20 °C. The methods of rEsxAB- and rHla_m_-specific IgA detection were mostly the same as previously described in Section 2.7, while the secondary antibody was HRP-conjugated goat anti-mouse IgA (Proteintech).

### 2.9. IFN-γ and IL-17 Levels in the Kidney and Spleen Tissue

IFN-γ and IL-17 levels in the tissue of BALB/c mouse kidneys and spleens were measured by ELISA. Then, 1 mL PBS solution was added to 0.1 g tissue. After homogenization and centrifugation, the supernatant was used for IFN-γ and IL-17 detection by double antibody sandwich method with the ELISA kit (R&D Systems, Minneapolis, MN, USA). Most practices were the same as antibody detection.

### 2.10. BALB/c Mice Challenged with S. aureus USA300

On day 14 after the booster immunization, all BALB/c mice were intravenous injected (i.v.) with *S. aureus* USA300 of 5 × 10^7^ CFU in 50 μL PBS solution. Additionally, they were finally sacrificed after 14-day continuous observation. Then, the abscess incidence, bacterial loading, and histopathological damage in the kidneys were evaluated.

### 2.11. Statistical Analysis

Data were analyzed with GraphPad Prism software and the R. Shapiro–Wilk test was used for testing normality. IgG and IgA levels were presented as the mean ± SD and analyzed by t-test. IFN-γ and IL-17 levels were presented as the mean ± SD and analyzed by one-way ANOVA and Tukey’s multiple-comparison test. Kruskal–Wallis test and Dunn’s multiple-comparison test were utilized for bacterial loading analysis and the proportion of abscess area analysis as part of the data did not meet the normal distribution.

## 3. Results

### 3.1. rEsxAB and rHla_m_ Synthesis in Recombinant Attenuated Regulated Delayed Lysis Salmonella Typhimurium Strain χ11802 and Secretion into the Medium

**Figure 1:**
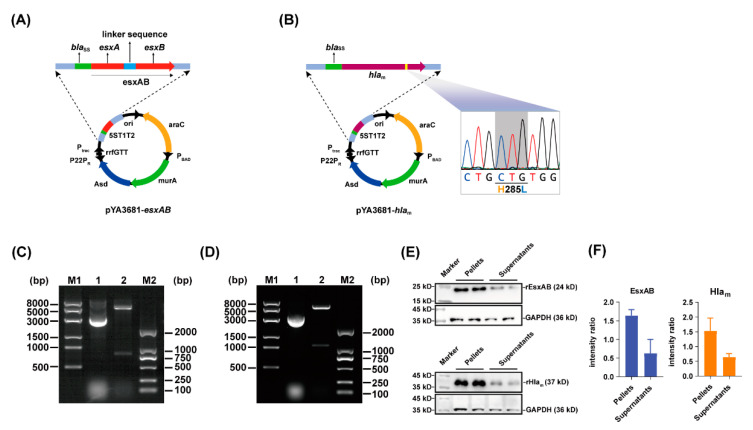
Construction of pYA3681−*esxAB* and pYA3681−*hla*_m_ plasmids and protein expression in recombinant strains of χ11802. (**A**) Construction scheme of pYA3681−*esxAB*. (**B**) Construction scheme of pYA3681−*hla*_m_. (**C**) The recombinant pYA3681−*esxAB* plasmid was identified by restriction endonuclease digestion. 8K DNA marker (Lane M1), pYA3681−*esxAB* plasmid (Lane 1), pYA3681−*esxAB* plasmid double-enzyme digested with *Nco*I and *Sma*I (Lane 2), and 2K DNA marker (Lane M2). (**D**) The recombinant pYA3681−*hla*_m_ plasmid was identified by restriction endonucleases. 8K DNA marker (Lane M1), pYA3681−*hla*_m_ plasmid (Lane 1), pYA3681−*hla*_m_ plasmid double-enzyme digested with *Nco*I and *Sma*I (Lane 2), and 2K DNA marker (Lane M2). (**E**) rEsxAB and rHla_m_ synthesis in recombinant strains of χ11802 and the recombinant proteins released in the supernatant culture medium were analyzed by Western blot. (**F**) Intensity ratio of EsxAB and Hla_m_ expression in recombinant strains of χ11802.

rEsxAB and rHla_m_ expression strains were constructed with regulated delayed lysis attenuated *Salmonella* Typhimurium strain χ11802. The schemes of recombinant plasmids are shown in the Figure 1A,B. Extracted from recombinant strains of χ11802, the plasmids were verified by double-enzyme digestion with *Nco*I and *Sma*I, and the *esxAB* (Figure 1C, Lane 2) and *hla*_m_ (Figure 1D, Lane 2) gene fragments were consistent with their expected length. The sequencing results further confirmed these (data not shown). The β-lactamase secretion signal peptide (coded by *bla*_SS_) was added to the N-terminal of the antigen proteins, which aims to transfer the proteins to the periplasmic space via the type II secretion system and eventually released to the outside [32]. Western blot showed that rEsxAB and rHla_m_ were synthesized in χ11802, and the recombinant proteins were released into the medium (Figure 1E,F).

### 3.2. The Survival of Recombinant Attenuated Regulated Delayed Lysis Salmonella Typhimurium Strains Strictly Dependent on Arabinose In Vitro and In Vivo

**Figure 2:**
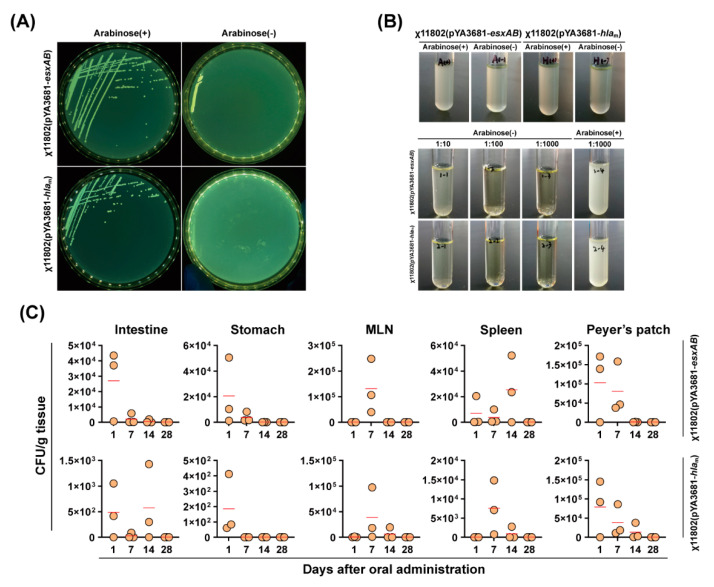
Recombinant strains of χ11802 survival status in vitro and in vivo in the absence of arabinose. (**A**) χ11802(pYA3681−*esxAB*) and χ11802(pYA3681−*hla*_m_) survival status on LB plate with or without 0.02% *w/v* arabinose. (**B**) χ11802(pYA3681−*esxAB*) and χ11802(pYA3681−*hla*_m_) survival status in LB broth with or without 0.02% *w/v* arabinose. (**C**) Bacterial loading of χ11802(pYA3681−*esxAB*) and χ11802(pYA3681−*hla*_m_) in mouse intestines, stomachs, MLNs, spleens, and Peyer’s patches over time, after oral administration, with the Mean value of the bacterial loading presented as red dash.

Recombinant attenuated regulated delayed lysis *Salmonella* Typhimurium strains required an exogenous source of arabinose for colonization and survival [28]. However, it was unclear whether this would be different or add side effects to the recombinant strains after *S. aureus* antigen genes were inserted into the pYA3681 plasmid. These were verified in three steps. Firstly, recombinant strains, χ11802(pYA3681−*esxAB*) and χ11802(pYA3681−*hla*_m_), were streaked on LB agar plate with or without 0.02% *w/v* arabinose. As a result, the LB agar plate with arabinose enabled the recombinant strains to grow well, while it showed growth inhibition on the arabinose-free LB agar plate (Figure 2A). This indicated that arabinose must be provided for recombinant strain growth. Secondly, considering that the recombinant strains would colonize in mice, we inoculated the recombinant strains of χ11802 into LB broth to determine growth restrictions in an arabinose-free liquid environment. The results showed that the recombinant strains could only be subcultured to the second generation in arabinose-free LB broth, compared with their growth status in LB broth with arabinose (Figure 2B). This provided a basis for the growth restriction of recombinant strains in the host. Thirdly, the BALB/c mice were orally administrated with recombinant strains to determine how long the recombinant strains would survive in vivo. On days 1, 7, 14, and 28 after oral administration, bacterial loading of the recombinant strains of χ11802 was evaluated. Although it showed that there were individual differences in mice, and recombinant strains migrated into MLN (mesenteric lymph nodes), spleen, and Peyer’s patch to a certain degree, the recombinant strains of χ11802 could be cleaned up on day 28 after oral administration. In the meantime, all vaccinated mice stayed in a good state.

### 3.3. Recombinant Attenuated Regulated Delayed Lysis Salmonella Typhimurium Strains Eliciting Serum IgG- and Intestinal Mucus IgA-Mediated Immune Responses 

**Figure 3:**
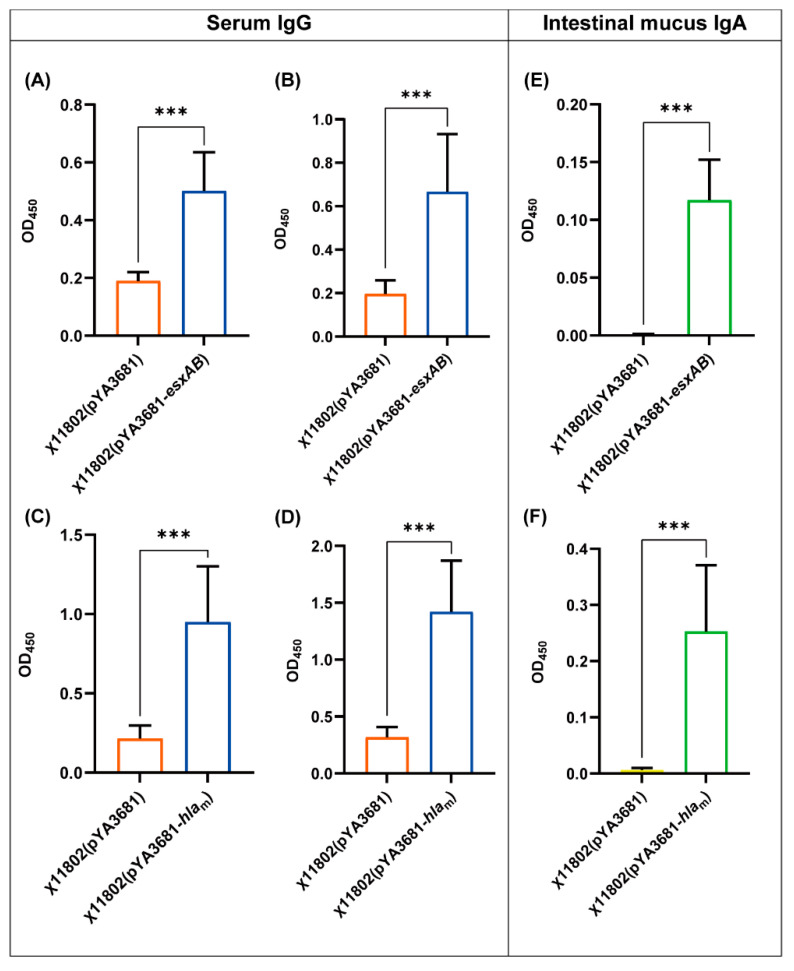
Antigen-specific IgG and IgA immune responses after BALB/c mice were orally administrated with recombinant strains of χ11802. In (**A**–**E**), mice were orally administrated with χ11802(pYA3681), χ11802(pYA3681−*esxAB*), or χ11802(pYA3681−*hla*_m_). (**A**,**B**) Serum anti-rEsxAB specific IgG level on day 14 after primary and booster immunization. (**C**,**D**) Serum anti-rHla_m_ specific IgG level on day 14 after primary and booster immunization. (**E**,**F**) Intestinal mucus anti-rEsxAB and anti-rHla_m_ specific IgA level on day 7 after booster immunization. *** *p* < 0.001.

To determine the humoral and mucosal immunity elicited by recombinant attenuated regulated delayed lysis *Salmonella* Typhimurium strains, the serum IgG and intestinal mucus IgA were evaluated by ELISA. On day 14 after primary and booster immunization, mouse serum was collected and rEsxAB- and rHla_m_-specific IgG were measured. The χ11802(pYA3681−*esxAB*)-vaccinated group and χ11802(pYA3681−*hla*_m_)-vaccinated group both showed that IgG level was significantly higher than the χ11802(pYA3681)-vaccinated group (Figure 3A–D). This indicated that orally administrated recombinant attenuated *Salmonella* Typhimurium strains successfully elicited humoral immunity. These vaccinated BALB/c mice (n = 10) were then challenged with *S. aureus* USA300.

On day 7 after the booster immunization, the remaining vaccinated BALB/c mice (n = 9) in each group were evaluated using mucus IgA. It showed that the IgA level was also significantly higher than that in the χ11802(pYA3681)-vaccinated group in the intestinal mucus of the χ11802(pYA3681−*esxAB*)-vaccinated group and χ11802(pYA3681−*hla*_m_)-vaccinated group, which suggested that mucosal immunity was elicited (Figure 3E,F). These kidneys and spleens of mice were then used for measuring IL-17 and IFN-γ levels.

### 3.4. IL-17 and IFN-γ Production Elicited by Recombinant Attenuated Regulated Delayed Lysis Salmonella Typhimurium Strains

**Figure 4:**
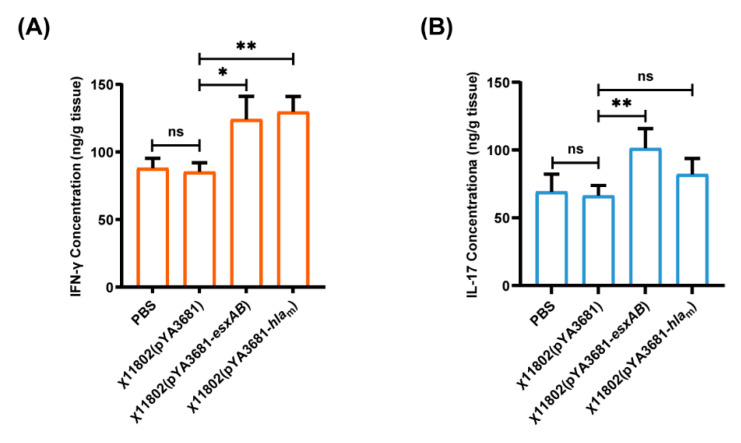
IFN-γ and IL-17 production in mouse kidney tissue elicited by oral administration with the recombinant strains of χ11802. In (**A**,**B**), mice were orally administrated with PBS solution, χ11802(pYA3681), χ11802(pYA3681−*esxAB*), or χ11802(pYA3681−*hla*_m_). (**A**) IFN-γ level in mouse kidney tissue. (**B**) IL-17 level in mouse kidney tissue. * *p* < 0.05; ** *p* < 0.01; ns, no significance.

In previous research, Th1- and Th17-mediated immunity were indicated to be very important for protection against *S. aureus* infection [14,33]. Therefore, the IL-17 and IFN-γ levels in the kidney and the spleen were measured. After booster immunization, the IL-17 and IFN-γ levels of the χ11802(pYA3681−*esxAB*)-vaccinated group were significantly higher than those of the χ11802(pYA3681)-vaccinated group in the kidney (Figure 4A,B). The IFN-γ level of the χ11802(pYA3681−*hla*_m_)-vaccinated group was also significantly higher than the χ11802(pYA3681)-vaccinated group. Regarding the production of IL-17 and IFN-γ in mouse spleen, they showed no significant increase (Figure S1A,B). These results indicated that Th1- and Th17-biased cellular responses were elicited after oral administration with χ11802(pYA3681−*esxAB*). Oral administration with χ11802(pYA3681− *hla*_m_) could elicit a Th1 cellular response while showing a weak effect for the Th17 cellular response.

### 3.5. Mouse Kidney Abscess Formation after Challenged with S. aureus USA 300

**Figure 5:**
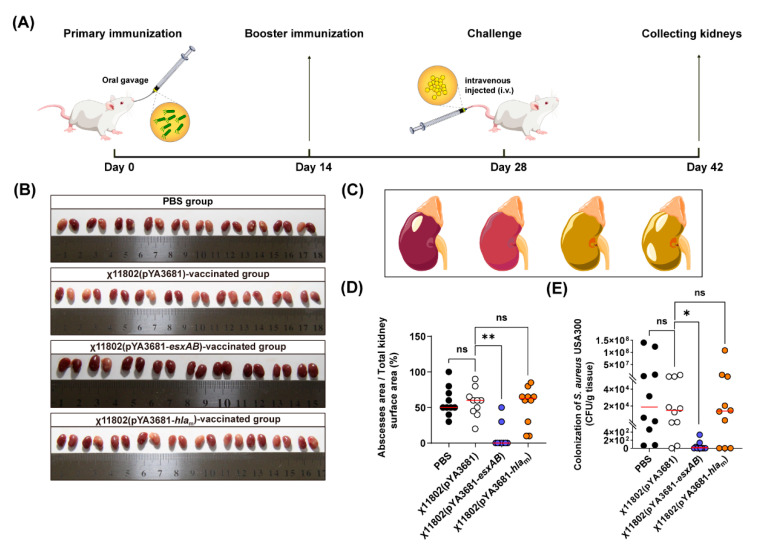
Abscess formation after BALB/c mice were challenged with *S. aureus* USA300 by i.v. (**A**) Immunization and challenge strategy. (**B**) Abscess formation of BALB/c mouse kidneys. (**C**) Four different degrees of kidney abscess symptoms. (**D**) Abscess area proportion of total kidney surface area. (**E**) Colonization of *S. aureus* USA300 in the kidneys after BALB/c mice challenge. * *p* < 0.05; ** *p* < 0.01; ns, no significance.

After challenge with *S. aureus* USA300 by i.v., all BALB/c mouse kidneys were collected on day 14 (Figure 5A,B). There were 10 mice in each vaccinated group initially, except 1 died in the χ11802(pYA3681−*esxAB*)-vaccinated group before challenged. The kidney abscess formation was evaluated, and the degree of abscesses was summarized into four patterns (Figure 5C). The abscess area proportion of the whole kidney surface was approximately estimated. As the results showed, the χ11802(pYA3681−*esxAB*)-vaccinated group presented a relatively low incidence of kidney abscess formation with a significant difference against the χ11802(pYA3681)-vaccinated group (Figure 5D), and the staphylococcal load was also significantly decreased in contrast with the χ11802(pYA3681)-vaccinated group (Figure 5E). However, the χ11802(pYA3681−*hla*_m_)-vaccinated group did not present an obvious inhibiting effect on abscess formation (Figure 5D,E). 

### 3.6. Kidney Tissue Lesion after BALB/c Mice Challenged with S. aureus USA 300

**Figure 6:**
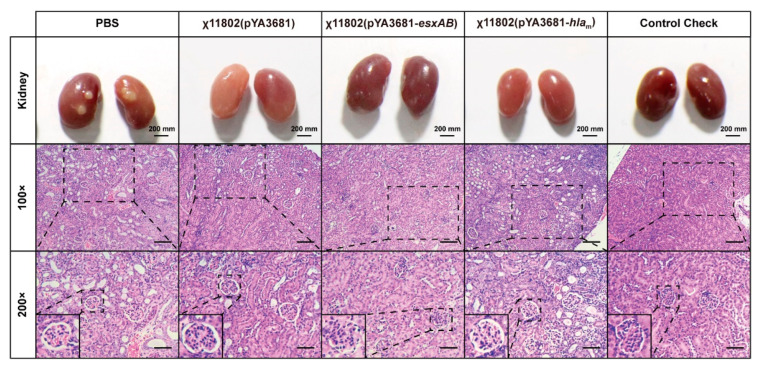
Histopathological evaluation of kidney after BALB/c mice were challenged with *S. aureus* USA300. Pathological section stained with H&E. The 100× magnification with a 50 μm bar. The 200× magnification with a 100 μm bar.

The degree of kidney tissue lesion was further evaluated by kidney pathology slides, which were stained with hematoxylin and eosin (H&E) (Figure 6). The kidney pathological sections of the mice without vaccination and challenge were used as the blank control (Control Check). All BALB/c mice showed a varying degree of kidney injury after challenge with *S. aureus* USA300. Among them, the kidney injury degree of the χ11802(pYA3681−*esxAB*)-vaccinated group was the least. The PBS group, the χ11802(pYA3681)-vaccinated group, and the χ11802(pYA3681−*hla*_m_)-vaccinated group all presented atrophy of kidney tubule epithelial cells, interstitial edema, and neutrophil infiltration. However, the χ11802(pYA3681−*esxAB*)-vaccinated group showed improvement.

## 4. Discussion

As an opportunistic pathogen, *S. aureus* causes a wide range of diseases. In most cases, antibiotics are utilized for the treatment of *S. aureus* infection. However, the multiple resistant strains reduce the effectiveness of the antibiotic. What is worse, the abscess formation will make it more difficult to cure [3]. Previous studies have mentioned that kidney abscess is an important symptom of *S. aureus* infection [4,5,6]. In this study, we attempted to develop a kind of protective vaccine to inhibit abscess formation and eventually prevent *S. aureus* infection. It has been confirmed that intracellular persistence makes *S. aureus* escape from professional phagocytes and extracellular antibiotics [34]. Therefore, we aimed to emphasize the cooperation of humoral immunity and cellular immunity in this study. 

The *S. aureus* antigens selected in the research were experimentally proved to be able to inhibit or moderate the abscess formation [11,12]. The Hla mutation (Hla_m_) was used as delivered antigen according to its immune protective effect in our previous experimental data. *S. aureus* EsxA and EsxB as subunit vaccines, utilized for immunization alone or together, were able to promote the induction of Th1- and Th17-biased immune responses [14]. However, the disadvantage of subunit vaccines is obvious: the proteins need to be extracted at a high cost. A low-cost and easy-to-use method is desired for immunization, which synthesizes and delivers antigens at the same time with no compromised immune protective effect. Live attenuated *Salmonella* Typhimurium seems to be a choice.

Live attenuated *Salmonella* Typhimurium has been developed as a mature delivery vector. As live oral vaccines, there has been meaningful progress in antibacterial [29,35], antiviral [36], antiparasitic [37,38], and antitumor [39] research. Though with good features as delivery vaccines, live attenuated *Salmonella* Typhimurium strains remain potential side effects. Meanwhile, there is no clear evidence showing how recombinant proteins will affect bacterial properties. Therefore, the dependence of the recombinant strains on arabinose was tested in vitro and in vivo. The results showed that arabinose was necessarily externally provided for the growth of recombinant strains (Figure 2A,B). This provided a basis that, after oral administration, the recombinant strains would be gradually cleaned up in the host. Further research showed that the recombinant strains migrated into MLN (mesenteric lymph nodes), spleen, and Peyer’s patch to a certain degree; however, eventually cleared up within 28 days after oral administration (Figure 2C). During observation, all the vaccinated BALB/c mice stayed in a good state. These indicated that oral administration with χ11802(pYA3681−*esxAB*) and χ11802(pYA3681−*hla*_m_) would not cause obvious damage to the host.

Humoral immunity and cellular immunity are important against pathogen invasions [40]. After oral administration with the recombinant strains, the humoral immune response was elicited at varying levels via the mucosal route in the host. rEsxAB- and rHla_m_-specific IgG in the serum, as well as IgA in the intestinal mucosa, were detected. In the early stage of *S. aureus* infection, specific antibodies and complement components in the serum play critical roles in the initiation of phagocytosis [41]. When *S. aureus* invades the extracellular, rEsxAB- and rHla_m_-specific IgG could be crucial for binding and neutralizing. However, in previous studies, *S. aureus* EsxA and EsxB as subunit vaccines, utilized for immunization individually or together elicited high titers of specific antigens, but they did not prevent infection, which other immune mechanisms must compensate for protection [14]. As research showed, Th1- and Th17-biased immune responses worked in *S. aureus* infection [42], and live attenuated *Salmonella* Typhimurium could elicit specific cellular immunity [28].

As previous studies have mentioned, *S. aureus* can invade host cells, and the intracellular *S. aureus* persists and replicates, some of which can enter the cytoplasm [34,43,44]. The intracellular *S. aureus* will release after phagocyte lysis and serves as a potential route for dissemination of infection [45]. Once *S. aureus* survives in phagocytes, to eliminate the intracellular pathogen, T helper cells, cytotoxic T cells, natural killer cells, and cytokine IFN-γ are required [46]. IFN-γ is the key cytokine to developing Th1-biased cellular immune responses [47]. Th17 cells could mediate serotype-independent protection, including recruiting neutrophils and macrophages to mucosal sites and activating B cell antibody responses [48]. Moreover, in the mucosal tissues, long-life Th17 effector memory cells had potential against pathogens [49]. In this study, IFN-γ and IL-17 in the kidney and spleen of BALB/c mice were evaluated after booster immunization. The IFN-γ level of kidney tissue in the χ11802(pYA3681−*esxAB*)-vaccinated group and the χ11802(pYA3681−*hla*_m_)-vaccinated group were significantly higher than that in the χ11802(pYA3681)-vaccinated group, and the IL-17 level of kidney tissue in the χ11802(pYA3681−*esxAB*)-vaccinated group was also significantly higher than that in the χ11802(pYA3681)-vaccinated group (Figure 4A,C). The previous study has reported that live attenuated *Salmonella* typhimurium vaccines delivering *S. aureus* antigens could elicit Th1- and Th17-biased cellular immune responses [50]. It could be inferred that, in this study, Th1/Th17 cellular immune responses could play essential roles in *S. aureus* infection. However, findings on IFN-γ and IL-17 levels in mouse spleen remained inconclusive in the kidneys, which was worth further exploration (Figure S1A,B).

After BALB/c mice were challenged with *S. aureus* USA300 by i.v., all of them were evaluated for kidney abscess formation on day 14 (Figure 5A,B). Among all the vaccinated groups, the χ11802(pYA3681−*esxAB*)-vaccinated group showed obvious inhibition in abscess formation. This is consistent with the subunit vaccine efficacy of EsxA and EsxB for inhibiting kidney abscess formation [12,14]. However, the χ11802(pYA3681−*hla*_m_)-vaccinated group did not show distinctive inhibition efficacy on abscess formation. As the protective effects of Hla were presented in murine pneumonia, χ11802(pYA3681−*hla*_m_) potential deserves further development.

This study showed that oral administration with χ11802(pYA3681−*esxAB*) and χ11802(pYA3681−*hla*_m_) could elicit humoral immunity and varying degrees of Th1/Th17 cellular immune response in the host. However, only χ11802(pYA3681−*esxAB*) could effectively inhibit abscess formation in the kidney. There are still several limitations that should be mentioned in this study. Firstly, only the *S. aureus* USA300 strain was used for the challenge experiment, and other serotypes of *S. aureus* should also be tested to obtain more common conclusions. Secondly, this study only showed the efficacy of recombinant attenuated regulated delayed lysis *Salmonella* Typhimurium strains as oral vaccines in inhibiting kidney abscess formation and some basic effects on the host immune system; the immune mechanism remains further studied.

In summary, our research showed the great potential of recombinant attenuated regulated delayed lysis *Salmonella* Typhimurium strains as oral vaccines inhibiting *S. aureus* abscess formation.

## 5. Conclusions

Oral administration with recombinant regulated delayed lysis attenuated *Salmonella* Typhimurium strain χ11802 delivering *S. aureus* EsxAB can elicit humoral, Th1- and Th17-mediated cellular immunity, and shows the significant inhibition of kidney abscess formation. This study provided experimental data and showed the great potential for the live oral vaccine development with regulated lysis attenuated *Salmonella* Typhimurium strains against *S. aureus* infection.

## Figures and Tables

**Table 1:** Bacterial strains and plasmids used in this study.

Strain or Plasmid	Description	Source or Reference
Strain		
χ6212	F^−^ λ^−^ φ80 Δ*(lacZYA-argF) endA1 recA1 hsdR17 deoR thi-1 glnV44 gyrA96 relA1* Δ*asdA4*	Genotype referenced Xin et al. [32]
χ11802	ΔP_murA25_::TT *araC* P_BAD_ *murA DasdA27*::TT *araC* P_BAD_*c2* Δ(*wza-wcaM*)-*8* Δ*pmi-2426* Δ*relA198*::*araC* P_BAD_ *lacI* TT Δ*recF126*	Genotype referenced Jiang et al. [29]
χ6212(pYA3681−*esxAB*)	χ6212 carrying pYA3681−*esxAB*	This study
χ6212(pYA3681−*hla*_m_)	χ6212 carrying pYA3681−*hla*_m_	This study
χ11802(pYA3681)	χ11802 carrying pYA3681	This study
χ11802 (pYA3681−*esxAB*)	χ11802 carrying pYA3681−*esxAB*	This study
χ11802 (pYA3681−*hla*_m_)	χ11802 carrying pYA3681−*hla*_m_	This study
*S. aureus* USA300		American Type Culture Collection (Manassas, VA, USA)
Plasmid		
pUC57−*esxAB*	pUC57::*bla*_SS_::HA-tag:: *esxAB*	GenScript Bio Co.
pUC57−*hla*_m_	pUC57::*bla*_SS_::HA-tag:: *hla*_m_	GenScript Bio Co.
pYA3681	P_trc_ pBR ori araC* P_BAD_ SD-GTG-*asdA* SD-GTG-*murA* P22 P_R_ antisense mRNA	Genotype referenced Jiang et al. [29]
pYA3681−*esxAB*	pYA3681::*bla*_SS_::HA-tag:: *esxAB*	This study
pYA3681−*hla*_m_	pYA3681::*bla*_SS_::HA-tag:: *hla*_m_	This study

## Data Availability

All data that this study is based upon are available from the corresponding author upon request.

## Supplementary Materials

The following supporting information can be downloaded at: https://www.mdpi.com/article/10.3390/vaccines10071073/s1, Figure S1: IFN-γ and IL-17 production in mouse spleen tissue elicited by oral administration with the recombinant strains of χ11802.
