# Supplementary material for: Oral Administration with Recombinant Attenuated Regulated Delayed Lysis Salmonella Vaccines Protecting against Staphylococcus aureus Kidney Abscess Formation
Source: Vaccines (Basel). 2022 Jul 4;10(7):1073. doi: 10.3390/vaccines10071073 (PMC9324569; doi:10.3390/vaccines10071073)
Supplement: Supplementary file 1 [file vaccines-10-01073-s001.zip › vaccines-1742852-supplementary.pdf]

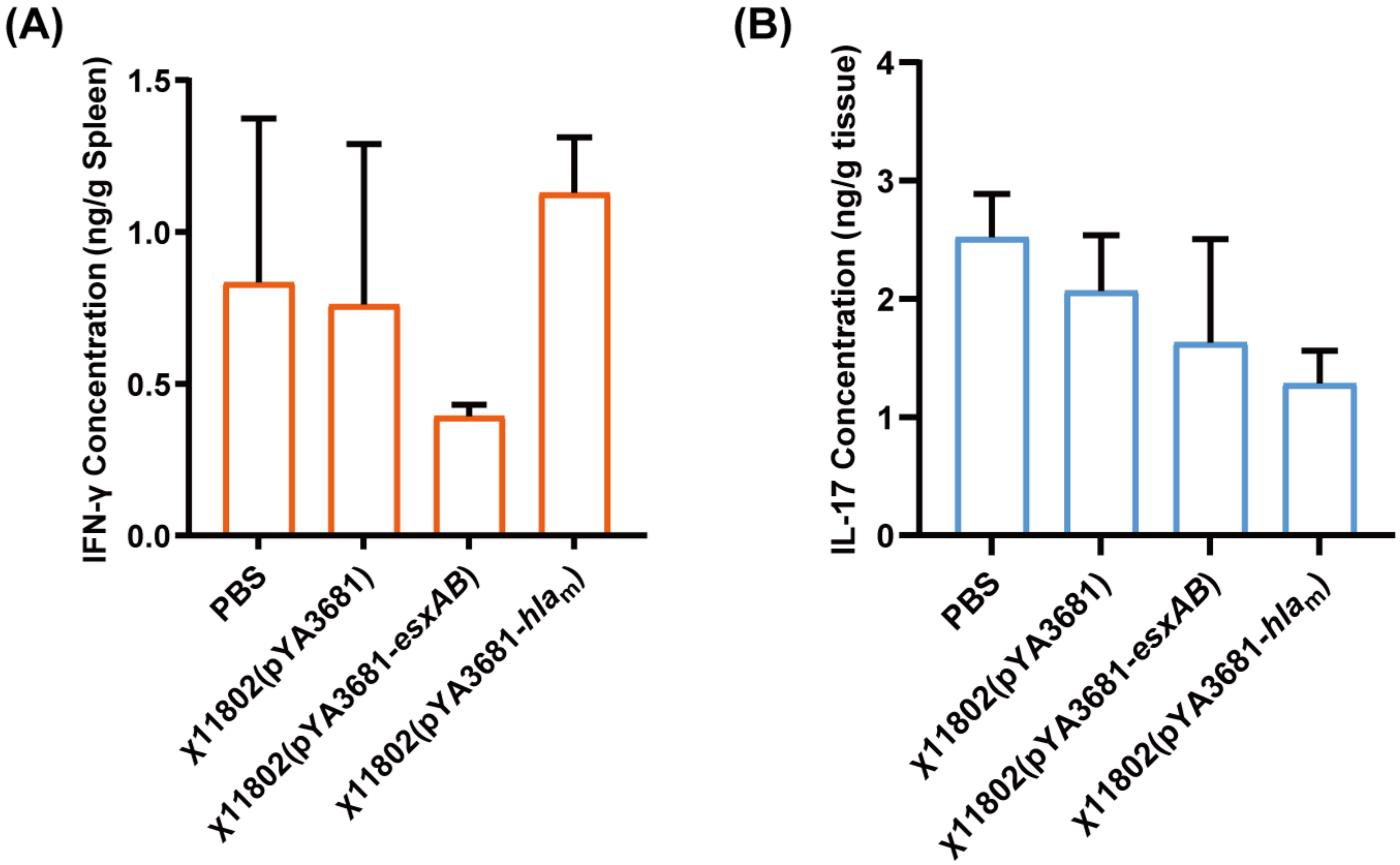

Figure S1: IFN-γ and IL-17 production in mouse spleen tissue elicited by oral administration with the recombinant strains of χ11802.
